# Clinical efficacy of low-intensity pulsed ultrasound plus hormone therapy in patients with postherpetic neuralgia: An observational study

**DOI:** 10.12669/pjms.41.7.11219

**Published:** 2025-07

**Authors:** Meng Li, Lingzhi Li, Chenqing Liu, Weili Ji

**Affiliations:** 1 Meng Li Department of Pain, Affiliated Hospital of Hebei University, Baoding 071000, Hebei, China; 2 Lingzhi Li Department of Pain, Affiliated Hospital of Hebei University, Baoding 071000, Hebei, China; 3 Chenqing Liu Department of Emergency, The No. 2 Hospital of Baoding, Baoding 071000, Hebei, China; 4 Weili Ji Department of Pain, Affiliated Hospital of Hebei University, Baoding 071000, Hebei, China

**Keywords:** Low-intensity pulsed ultrasound, Glucocorticoid, Postherpetic neuralgia, Treatment

## Abstract

**Objective:**

To evaluate the clinical efficacy of low-intensity pulsed ultrasound (LIPUS) plus hormonal therapy in treating patients with postherpetic neuralgia (PHN).

**Method:**

This was an observational study. A total of 120 patients with diabetic peripheral neuropathic pain treated at Affiliated Hospital of Hebei University from May 2023 to May 2024 were selected and randomly divided into two groups. The control group received standard treatment, including maintaining the cleanliness and dryness of the rash, wearing loose clothing, local ultraviolet or infrared irradiation. The study group received LIPUS plus hormone therapy in addition to the standard treatment. Clinical outcomes, pain intensity, sleep quality and anxiety status before and after treatment were compared between the two groups.

**Results:**

The overall response rate was 88.33% in the study group and 70.00% in the control group, with the study group showing significantly higher efficacy (p= 0.013). There were no significant differences in VAS and PSQI scores between the two groups before treatment(p>0.05, respectively). After treatment, the study group exhibited more pronounced reductions in the VAS and PSQI scores compared to the control group, with statistically significant differences between the two groups (p< 0.05, respectively). There was no significant difference in the SAS score between the two groups before treatment (p= 0.556).

**Conclusion:**

LIPUS plus hormone therapy may provide significant clinical benefits in treating PHN, with marked improvements in pain relief, without increasing the risk of adverse reactions. Therefore, this combination therapy is considered safe and efficacious for patients with PHN.

## INTRODUCTION

Herpes zoster is a neuroinflammatory condition caused by the varicella-zoster virus, which shows a strong affinity for the nervous system. After an initial infection, if the individual’s immune system is robust, the virus can remain dormant in the dorsal root ganglia of the spinal cord or sensory ganglia of cranial nerves. When immunity weakens, the dormant virus reactivates, damaging nerves and migrating along them to the skin, where lesions form, often accompanied by significant neuralgia.^1^ Some patients experience residual nerve pain, known as postherpetic neuralgia (PHN). PHN, a form of persistent pain typically characterized by allodynia and hyperalgesia, is one of the harmful outcomes that patients may experience after the acute phase of herpes zoster resolves.

The pain caused by PHN can severely affect patients’ quality of life, sleep quality and ability to engage in daily activities.^2^ It is the most serious complication of herpes zoster and when pain persists for one month or more after the healing of skin lesions, it can induce psychosocial dysfunction and negatively impact patients’ quality of life.^3^ PHN is classified as neuropathic pain, with mechanisms involving central and peripheral sensitization.^4^ Despite advances in treatment, PHN remains difficult to cure. Current clinical treatments for PHN primarily include oral medications, such as anticonvulsants and antidepressants, as well as local therapies like nerve blocks, spinal cord stimulation and radiofrequency treatments, which often involve agents such as lidocaine and capsaicin.

However, oral medications tend to have side effects that make long-term adherence difficult for patients, while invasive methods require careful monitoring of their efficacy, safety and tolerability.^5^ Moreover, these procedures are complex, limiting their clinical use. In this study, LIPUS was employed in combination with glucocorticoids to treat PHN, evaluate the clinical efficacy of LIPUS plus hormonal therapy in treating patients with PHN.

## METHODS

This was an Observational Study. A total of 120 patients with PHN admitted to Affiliated Hospital of Hebei University from May 2023 to May 2024 and randomly divided into two groups, with 60 patients in each group. According to the data of each indicator in the pre-survey, the sample size is estimated by 95% confidence interval and the largest one is the sample size of the study. The sample size required for each group was > 60 cases on the basis of Fisher exact probability. In the study group, there were 38 males and 22 females, aged between 33 and 62 years, with an average age of 50.30 ± 6.67 years. In the control group, there were 35 males and 25 females, aged between 31 and 64 years, with an average age of 51.55 ± 8.21 years. There were no significant differences in general patient information between the two groups, making them comparable (Table-I).

**Table-I:** Comparison of general patient information between the two groups (*χ̅*±*S*, n = 60).

Item	Study group	Control group	t/χ^2^	p
Age (years)	50.30±6.67	51.55±8.21	0.915	0.362
Male (n, %)	38 (63.33%)	35 (58.33%)	0.034	0.853
Disease duration (months)	4.37±1.23	4.80±1.36	1.825	0.0.070
VAS score	7.33±1.22	7.52±0.95	0.921	0.359
***Affected area (n, %)***				
Intercostal nerve	31 (51.67%)	34 (56.67%)	0.302	0.583
Brachial plexus	18 (30.00%)	16 (26.67%)	0.164	0.685
Lumbar nerve	11 (18.33%)	10 (16.67%)	0.058	0.810
***Underlying condition(s) (n, %)***				
Hypertension	6 (10.00%)	7 (11.67%)	0.086	0.769
Hyperlipidemia	8 (13.33%)	6 (10.00%)	0.323	0.570
Diabetes	13 (21.67%)	11 (18.33%)	0.208	0.648

*p >* 0.05

### Ethical Approval:

The study was approved by the Institutional Ethics Committee of Affiliated Hospital of Hebei University (No.: HDFYLL-KY-2023-090; Dated: September 25, 2023) and written informed consent was obtained from all participants.

### Inclusion criteria:

- A confirmed history of herpes zoster.
- Persistent pain lasting more than one month after the resolution of herpes zoster skin lesions.
- Moderate to severe pain (scoring > 4 on the Visual Analog Scale [VAS]) affecting work, daily life and sleep.
- First onset of the disease, with no prior pain treatment in the month before enrollment.
- Complete clinical data.
- Normal cognitive, mental and language abilities, enabling accurate description of pain changes under the guidance of a physician.
- Good treatment compliance from the patient and their family, willing and able to cooperate throughout the study.
- No contraindications to the medications used in this study.
- No local skin damage or lesions and able to undergo ultrasound therapy.
- Signed informed consent.

### Exclusion criteria:

- Severe organic diseases, such as abnormal liver or kidney function, hematological disorders, or neurological conditions.
- Allergic constitutions or a history of drug allergies.
- Consciousness disorders or mental abnormalities that hinder the completion of the study;
- Pregnancy or cancer.
- Intolerance to the medications or pulsed therapy used in this study.
- Recent use of medications that could affect the study, such as immunosuppressants or hormones.

### Treatment methods:

The control group received standard treatment, which included maintaining cleanliness and dryness of the rash, wearing loose clothing, local ultraviolet or infrared irradiation and administration of antiviral and analgesic medications. The study group, in addition to the standard treatment, underwent LIPUS and hormone therapy. The treatment protocol is described as follows: For LIPUS therapy, the treatment dose ranged from 0.5 to 1.0 W/cm². Direct external scans were used, with iodinated glycerin as the coupling agent.

The ultrasound probe was placed directly on the skin at the pain site and moved evenly in a spiral motion. The treatment duration was adjusted based on the size of the affected area, with a maximum of 20 min per session, once daily. Every 10 sessions constituted one course of treatment and a total of three courses were given. As to hormone therapy, prednisone was administered orally with the following regimen: 30 mg/d for the first three days, 20 mg/d for the next three days and 10 mg/d for the subsequent three days, then reduced to five mg/d. Each course lasted 12 days, with a total of two courses given.

### Outcome measures:

### Clinical efficacy evaluation:

The clinical efficacy of both groups was evaluated after four weeks of treatment. The outcomes were classified as follows: Complete response (CR): complete disappearance of pain; substantial response (SR): significant pain relief, with only occasional, tolerable symptoms; partial response (PR): moderate pain relief, with occasional symptoms that require medication; no response (NR): no improvement or worsening of symptoms. The overall response rate (ORR) was calculated as: ORR = (Number of CR cases + Number of SR cases + Number of PR cases) / Total number of cases × 100%.

### Pain assessment:

The self-reporting VAS was used to evaluate the pain levels of the two groups before treatment and at 1, 2, 3 and 4 weeks after treatment. The scale ranges from 0 to 10, with lower scores indicating less pain (0 indicates no pain).

### Sleep quality assessment:

Sleep quality was evaluated using the Pittsburgh Sleep Quality Index (PSQI), with a score of 0 indicating no effect on sleep and a score of four suggesting inability to fall asleep.^6^

### Negative emotion assessment:

After treatment, both groups were assessed using the Self-Rating Anxiety Scale (SAS). Higher scores reflect more severe negative emotions. The SAS score is interpreted as follows: 50-60: mild anxiety; 61-70: moderate anxiety; and > 70: severe anxiety.

### Incidence of adverse reactions:

The incidence of adverse reactions in both groups was compared during the 4-week treatment period. The maximum follow-up time for patients in both groups was three months. And the cut-off time point was on May 2024.

### Statistical analysis:

This performed using SPSS 20.0. Measurement data were represented by (*χ̅*±*S*). Independent sample t-tests were used for between-group comparisons, while paired t-tests and variance analysis were applied for within-group comparisons. The chi-square (*χ*^2^) test was used to compare rates. A *p*-value of <0.05 was considered statistically significant.

## RESULTS

The comparison of treatment efficacy between the two groups is shown in Table-II. The results indicate that the ORR was 88.33% in the study group, significantly higher than 70.00% in the control group (*p* = 0.013). There was no significant difference in VAS scores between the two groups before treatment (*p* = 0.359). After four weeks of treatment, both groups showed a significant reduction in VAS scores (*p* = 0.000, respectively). Notably, the study group demonstrated a more pronounced decrease in VAS scores at 1, 2, 3 and 4 weeks compared to the control group (*p* = 0.000, respectively) (Table-III).

**Table-II:** Comparison of clinical efficacy between the two groups (*χ̅*±*S*, n = 60).

Group	CR	SR	PR	NR	ORR*
Study group	11	32	10	7	53 (88.33%)
Control group	6	28	8	18	42 (70.00%)
*χ^2^*					6.114
*p*					0.013

* p < 0.05

**Table-III:** Comparison of VAS scores before and after treatment between the two groups (*χ̅*±*S*, n = 60).

Group	Before treatment*	At 1 week post-treatment*	At 2 weeks post-treatment*	At 3 weeks post-treatment*	At 4 weeks post-treatment*	F	p
Study group*	7.33±1.22	6.03±1.07	4.45±1.03	2.03±0.76	1.27±0.45	445.171	0.000
Control group*	7.52±0.95	6.58±0.89	5.10±0.86	3.78±0.87	3.23±0.67	272.180	0.000
*t*	0.921	3.058	3.752	11.781	18.866		
*p*	0.359	0.003	0.000	0.000	0.000		

* p < 0.05

No significant difference was observed in PQSI scores between the two groups before treatment (*p* = 0.68). After four weeks of treatment, PQSI scores were significantly reduced in both groups (*p* = 0.00, respectively). Additionally, the study group exhibited a more pronounced reduction at 1, 2, 3 and 4 weeks compared to the control group (*p* = 0.00, respectively) (Table-IV). There was no significant difference in SAS scores between the two groups before treatment (*p* = 0.556). However, after treatment, SAS scores were significantly reduced in both groups (*p* = 0.000, respectively). Moreover, the study group showed a more significant reduction compared to the control group (*p* = 0.000, respectively) (Table-V). The incidence of adverse drug reactions after treatment was 20.00% in the study group and 13.33% in the control group, with no statistically significant difference (*p* = 0.327) (Table-III).

**Table-IV:** Comparison of PQSI scores before and after treatment between the two groups (*χ̅*±*S*, n = 60).

Group	Before treatment*	At 1 week post-treatment*	At 2 weeks post-treatment*	At 3 weeks post-treatment*	At 4 weeks post-treatment*	F	p
Study group*	3.45±0.50	1.88±0.49	1.23±0.53	0.87±0.43	0.53±0.50	328.172	0.000
Control group*	3.43±0.50	2.65±0.58	1.68±0.60	1.27±0.63	1.02±0.60	180.454	0.000
*t*	0.182	7.842	4.360	4.042	4.798		
*p*	0.856	0.000	0.000	0.000	0.000		

* *p <* 0.05

**Table-V:** Comparison of SAS scores before and after treatment between the two groups (*χ̅*±*S*, n = 60).

Group	Before treatment	After treatment*	t	p
Study group*	63.72±3.42	51.25±3.82	18.856	0.000
Control group*	64.08±3.38	58.60±3.57	8.636	0.000
*F*	0.591	10.893		
*p*	0.556	0.000		

* *p <* 0.05

## DISCUSSION

In our study, the ORR of LIPUS plus corticosteroid therapy for PHN was 88.33%, significantly higher than that of the control group (*p* = 0.013). After treatment, the study group showed significant reductions in VAS, PQSI and SAS scores compared to the control group and the differences were statistically significant (*p* < 0.05, respectively). The incidence of adverse reactions was 20.00% in the study group and 13.33% in the control group, with no statistically significant difference between the two groups (*p* = 0.327). However, recent studies suggest that corticosteroids reduce antibody production and inhibit the chemotaxis of inflammatory cells, thereby protecting affected cells from damage. This leads to the rapid reduction of congestion, edema and necrosis in the ganglia and corresponding sensory nerve fibers, preventing adhesions and reducing the risk of developing PHN.^7^ Ghanavatian et al.^8^ viewed that the use of corticosteroids combined with antiviral drugs has no side effects and does not interfere with the formation of specific immunoglobulins. Liu et al.^4^ suggested that low-dose, short-term corticosteroid therapy not only does not increase the risk of infection but also provides effective pain relief for PHN.

**Table-VI:** Comparison of post-treatment adverse drug reactions between the two groups (*χ̅*±*S*, n = 60).

Group	Thrombocytopenia	Hepatic/Renal dysfunction	Fever	Leukopenia	Gastrointestinal discomfort	Incidence rate*
Study group	1	3	0	4	4	12 (20.00%)
Control group	0	3	1	0	4	8 (13.33%)
*χ* ^2^						0.960
*p*						0.327

* *p >* 0.05

Herpes zoster, caused by the varicella-zoster virus, affects approximately one million people annually in the United States, with an individual’s lifetime risk estimated at 30%. For immunocompromised individuals, the incidence of herpes zoster increases by 20 to 100 times.^1^ Postherpetic neuralgia (PHN) develops in over 50% of patients with herpes zoster.^9^ Treatment options such as oral medications and minimally invasive interventions can provide short-term relief, but long-term efficacy is often difficult to sustain and relapses are common.^10^ The pathogenesis of PHN is complex. It is believed that acute-phase viral damage to the ganglia, if not adequately controlled, leads to a severe and prolonged inflammatory response, resulting in collagen deposition and scar tissue formation.^11^ The damage caused by the virus induces neurochemical, physiological and anatomical changes in neurons, which enhances the sensitivity of peripheral nociceptors, amplifying the pain signals they transmit.

This results in increased spontaneous neuronal firing, expansion of receptive fields, lowered thresholds for external stimuli and heightened responses to suprathreshold stimuli - key pathophysiological processes involved in PHN. The sustained amplification of these signals can further alter the structure and function of the spinal and supraspinal nervous systems, leading to central sensitization, which explains the clinical manifestations of spontaneous pain, hyperalgesia and allodynia in PHN.^12^ As the inflammatory response persists, degeneration and necrosis of afferent nerve fibers occur, leading to “deafferentation” of central neurons. This deafferentation causes secondary hyperexcitability of central neurons, creating a positive feedback loop that further intensifies the pain signals. This phenomenon is a likely pathophysiological mechanism of PHN and the root cause of persistent, intractable pain.^13^

The development of PHN is closely associated with neuroinflammation.^14^ The increase in inflammatory markers contributes to the exacerbation and progression of pain. Huang et al.^15^ suggested that LIPUS can upregulate the expression of nerve cell proliferation-related genes, thereby promoting nerve cell repair and regeneration. Aiyer et al.^16^ found that LIPUS is highly effective in treating pain. LIPUS exerts therapeutic effects by suppressing inflammatory cytokine levels, increasing antioxidant molecules, relieving pain, stimulating cell proliferation and differentiation and promoting tissue healing, angiogenesis and regeneration.

The benefits of LIPUS therapy are particularly evident in treating chronic inflammation.^17^ Xia et al.^18^ reported that LIPUS reduces inflammation and improves local antioxidant levels, creating a favorable microenvironment for nerve regeneration and facilitating nerve cell repair. Ultrasound therapy at appropriate intensities can enhance nerve cell vitality and proliferation, making it an effective, cost-efficient and non-invasive treatment option for patients with chronic pain. The use of corticosteroids remains controversial. It was previously believed that corticosteroids could suppress immune function, leading to viral dissemination and worsening of rashes.^19^

### Limitations:

First, it has a small sample size and lacks follow-up data. Second, it only compared the proposed combination therapy with traditional oral medication and did not include comparisons with minimally invasive treatments. We are working on expanding the sample size and actively collecting follow-up data. In future studies, we will also include minimally invasive treatment options. This will enable a more objective and accurate assessment of the advantages and disadvantages of this treatment approach.

## CONCLUSIONS

LIPUS plus corticosteroid therapy may provide significant pain relief for patients with PHN, with notable improvements in sleep quality and anxiety levels, without a significant increase in adverse reactions. Therefore, this combination therapy is considered safe and efficacious for treating PHN.

### Authors’ Contributions:

**ML** and **WJ:** Conceived and designed the study.

**LL:** Collected the data and performed the analysis. Critical Review.

**CL:** Writing of the manuscript and is responsible for the integrity of the study.

All authors have read and approved the final manuscript.
